# Minocycline reduces alveolar bone loss and bone damage in Wistar rats with experimental periodontitis

**DOI:** 10.1371/journal.pone.0309390

**Published:** 2024-10-04

**Authors:** Deborah Ribeiro Frazão, José Mario Matos-Souza, Vinicius Ruan Neves dos Santos, Rayssa Maite Farias Nazario, Victoria dos Santos Chemelo, Leonardo Oliveira Bittencourt, Gabriela de Souza Balbinot, Fabrício Mezzomo Collares, Walace Gomes-Leal, Railson Oliveira Ferreira, Cassiano Kuchenbecker Rösing, Alexandru Movila, Rafael Rodrigues Lima

**Affiliations:** 1 Laboratory of Functional and Structural Biology, Institute of Biological Sciences, Federal University of Pará (UFPA), Belém, Para, Brazil; 2 Dental Materials Laboratory, School of Dentistry, Federal University of Rio Grande do Sul (UFRGS), Porto Alegre, Rio Grande do Sul, Brazil; 3 Laboratory of Experimental Neuroprotection and Neuroregeneration, Institute of Collective Health, Federal University of Western Pará (UFOPA), Santarém, Para, Brazil; 4 Department of Periodontology, School of Dentistry, Federal University of Rio Grande do Sul (UFRGS), Porto Alegre, Rio Grande do Sul, Brazil; 5 Department of Biomedical Sciences and Comprehensive Care, Indiana University School of Dentistry, Indianapolis, Indiana, United States of America; 6 Indiana Center for Musculoskeletal Health, Indiana University School of Medicine, Indianapolis, Indiana, United States of America; Hamadan University of Medical Sciences, ISLAMIC REPUBLIC OF IRAN

## Abstract

This study aimed to investigate the impact of minocycline on the alveolar bone in experimental periodontitis in rats. Thirty Wistar rats were randomly assigned to three groups: control without periodontitis; experimental periodontitis induced by ligature; experimental periodontitis + intraperitoneal administration minocycline for seven days. Ligatures remained in place in both periodontitis groups for 14 days. At the end of the experiment, the animals were euthanized and one hemimandible underwent micro-computed tomography (micro-CT) analysis to assess vertical bone loss and alveolar bone quality. Histopathological analysis was performed on the other hemimandible. Statistical analysis was performed using ANOVA with Tukey’s post-test (p<0.05). The results showed a significant reduction in vertical bone loss in the animals treated with minocycline compared with untreated animals. Minocycline also preserved the alveolar bone thickness, number, spacing, and bone volume to tissue volume ratio. Histopathological analysis indicated that minocycline reduced bone resorption, decreased inflammatory response, and maintained the bone collagen fibers. This study demonstrated the effectiveness of minocycline in reducing vertical bone loss and preserved bone quality in rats with experimental periodontitis. The results of this study indicate that minocycline has the potential to serve as an additional treatment option for periodontitis. However, further research is warranted to assess the efficacy and safety of minocycline use in patients with periodontitis.

## Introduction

Periodontitis is a complex inflammatory condition that impacts the tissues responsible for supporting the teeth, which are the periodontal ligament, cementum, and alveolar bone, and is considered the 6^th^ most common inflammatory disease worldwide [1]. Periodontitis is a multifactorial inflammatory disease triggered by dental biofilms. In addition to oral biofilms, in the causal chain of periodontal diseases are other behavioral, environmental, and genetic factors that can potentially influence the beginning and development of periodontitis [2]. Furthermore, the immune host response plays a crucial role in determining the onset, progression, and, consequently, prognosis of patients affected by periodontal disease [3,4].

The treatment approach for periodontitis involves a stepwise strategy tailored to the disease stage, emphasizing personalized care plans and patient education [5]. The initial step focuses on motivating behavior change by addressing supragingival dental biofilm control, optimizing oral hygiene practices, and considering adjunctive therapies for gingival inflammation [6]. This step also involves risk factor control, including smoking cessation, better diabetes management, and lifestyle modifications [6]. The second step, cause-related therapy, targets subgingival biofilm and calculus removal through subgingival instrumentation. It may involve adjunctive physical or chemical agents or host-modulating agents. This step applies to all patients with periodontitis, especially in teeth with noticeable loss of periodontal support, including increased probing depth of the sulcus/pocket. The third therapy step is reserved for areas where the second step has not yielded satisfactory results. Interventions may include repeated subgingival instrumentation, access flap, ressective periodontal surgery, and ultimately regenerative procedures [5].

Regarding adjuvant therapies, tetracyclines have previously been employed to support periodontal treatment [7]. They are widely recognized for their extensive antimicrobial activity, encompassing various Gram-positive and Gram-negative bacteria, spirochetes, obligate intracellular bacteria, and even protozoan parasites [7]. Tetracyclines are typically categorized as bacteriostatic antibiotics, operating by interfering with ribosomal functions, specifically disrupting the coupling of aminoacyl transfer RNA (tRNA) during the elongation phase of translation [8]. However, the effectiveness of tetracycline has been challenged especially by the growing prevalence of resistance mechanisms [9]. Moreover, there are some risks associated with the use of tetracyclines during pregnancy [10]. This antibiotic crosses the placental barrier, exposing the fetus to potential adverse effects [11]. Additionally, human studies indicate that exposure during the second and third trimesters increases the incidence of dental discoloration and enamel hypoplasia in primary teeth [10,12].

To address the limitations associated with tetracyclines, minocycline, a second-generation tetracycline [13], has emerged as a viable alternative due to its improved efficacy against both gram-negative and gram-positive bacteria. Minocycline exhibits enhanced ribosomal affinity, resulting in a lower rate of bacterial resistance compared to other tetracyclines, along with potent anti-inflammatory properties [9,14].

Furthermore, similar to other tetracyclines, minocycline presents anti-inflammatory properties and exerts modulatory effects on the biology of periodontal fibroblasts, in addition to its efficiency against plaque microorganisms [13–17]. Notably, minocycline has demonstrated a high affinity for mineralized tissues, with positive effects on bone regeneration. In fact, tetracyclines have been recognized as one of the few classes of antibacterial agents with beneficial effects on bone tissue remodeling and healing processes [15].

The potential therapeutic effects of minocycline in bone physiology have been extensively studied to address various bone-related conditions [18]. The findings of a research investigation conducted on rats demonstrated that the systemic administration of minocycline hydrochloride resulted in a notable acceleration and enhancement of vertical bone augmentation during guided bone augmentation procedures. These findings provide additional evidence to substantiate the potential beneficial impact of minocycline on the structure of alveolar bone, thereby emphasizing its noteworthy contribution to the process of bone tissue regeneration [15].

Hence, the primary objective of this study was to thoroughly investigate the impact of minocycline on the alveolar bone of rats with ligature-induced experimental periodontitis. We aimed to understand minocycline’s potential in supporting bone regeneration and its influence on periodontal tissue dynamics in experimental periodontitis. Through thorough examination and analysis, we sought to expand our knowledge of minocycline’s therapeutic potential in managing periodontal diseases, particularly focusing on the alveolar bone.

## Methodology

### Study design and sample size estimation

This is a parallel, controlled, 3-arm, examiner-blind animal model study. The determination of the sample size was conducted using the G*Power software (Statistical Power Analyses 3.1.9.2), based on the study conducted by Castro et al. (2020) as a reference, considering an effect size of 1.893, an error probability of 0.05, and a power of 0.95. This estimation revealed a minimum number of 9 animals per group.

### Animals

A total of 30 male Wistar rats, aged 90 days, were included in this study. They were housed in individual cages within an animal facility at the Federal University of Pará. Animals were provided with a controlled diet consisting of 120g of food per animal and had unrestricted access to water. The study was conducted under a 12-hour light/dark cycle, with lights switched on at 7 a.m. The environmental temperature was maintained at 25°C constantly. Ethical approval for all procedures was obtained from the Ethics Committee on Experimental Animals of the Federal University of Pará, with protocol identifier 7599261120. The study strictly adhered to the guidelines outlined in the Guide for the Care and Use of Laboratory Animals [19], and it followed the ARRIVE recommendations for experimental procedures [20] and was assisted by the Preferred Reporting Items for Animal Studies in Endodontology, with adaptations for periodontology [21].

### Experimental procedures

The researchers randomly assigned the animals to three groups: a control group (C) consisting of animals without periodontitis and no use of minocycline (n = 10); an experimental periodontitis group (EP, n = 10), in which animals were subjected to ligature-induced periodontitis; and an experimental periodontitis with minocycline therapy group (EP+M, n = 10). On the first day of the experiment, the EP and EP+M groups underwent ligature-induced periodontitis. The study focused solely on examining the impact of minocycline on the occurrence and progression of periodontitis.

### Induction of experimental periodontitis

During the initial day of the experiment, all animals underwent anesthesia using xylazine hydrochloride (8 mg/kg) and ketamine hydrochloride (75 mg/kg). Our research team induced periodontitis by attaching a cotton thread around the cervical region of the mandibular first molars. The ligature remained in place for 14 days until euthanasia. We selected this duration based on the presence of a chronic injury pattern characterized by significant bone loss, as demonstrated by previous experiments conducted by our research team. [22–25].

### Minocycline administration

Seven days after inducing the lesion, the animals in the experimental periodontitis group with minocycline treatment received an intraperitoneal (i.p.) dose of minocycline (Sigma-Aldrich, USA) at 50 mg/kg every 12 hours for two days, followed by 25 mg/kg every 24 hours for five days. This dose has been used in previous studies from our research group and has been effective in inflammatory disorders in the central nervous system [14,26]. The animals in the remaining groups received sterile 0.9% saline solution i.p. for seven days.

**Fig 1:**
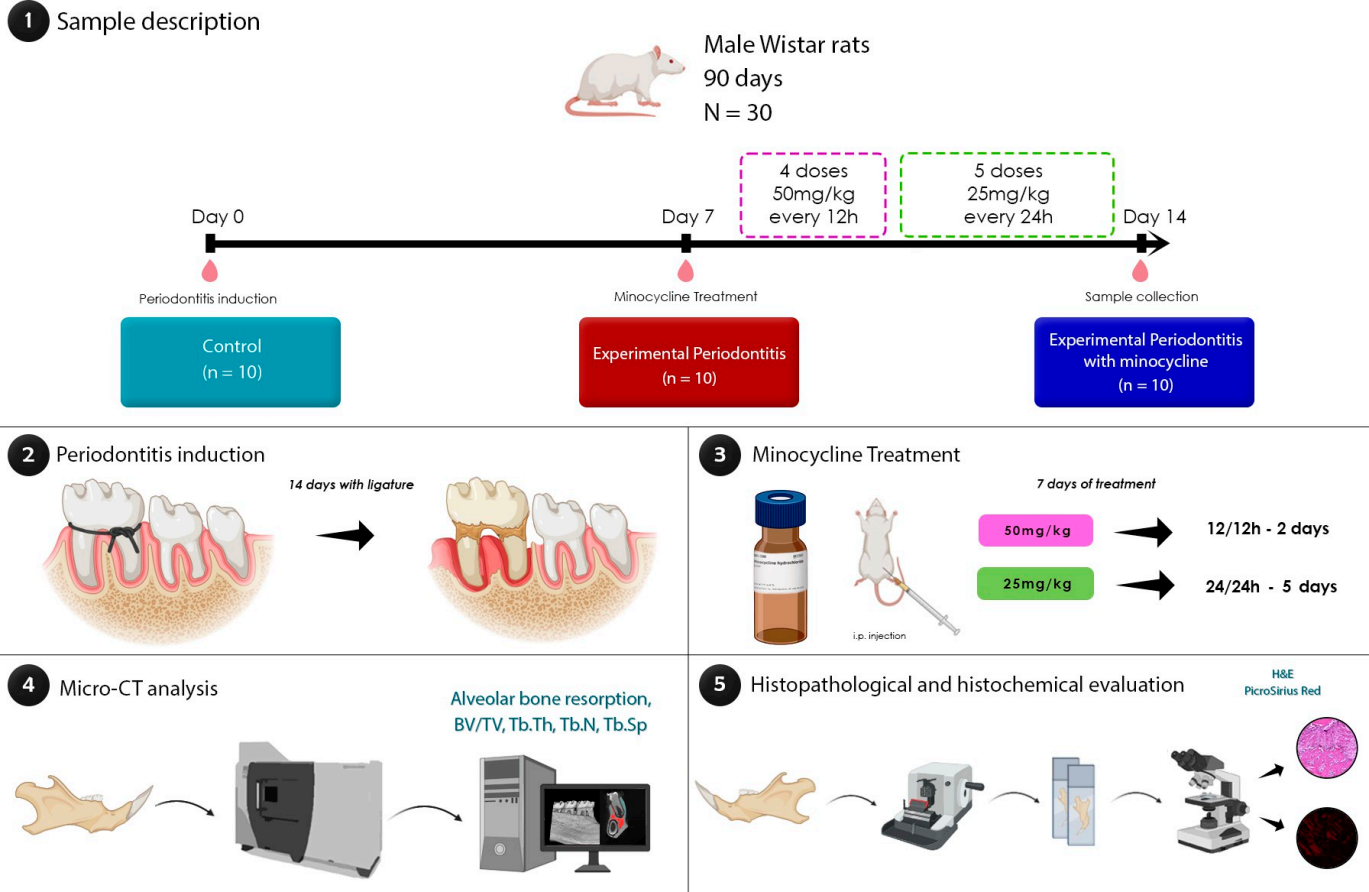
The image illustrates the five steps of the experiment. In the Sample description section (Step 1), thirty male Wistar rats, aged 90 days, were divided into three groups: Control (C), Experimental periodontitis without treatment (EP), and Experimental periodontitis with minocycline administration (EP+M). The study spanned 14 days, beginning with periodontitis induction on Day 0 (Step 2). On Day 7, the EP+M group began receiving minocycline, administered at 50 mg/kg every 12 hours for 2 days and 25 mg/kg every 24 hours for 5 days (Step 3). Micro-CT imaging was conducted (Step 4) to analyze bone loss and bone quality. Finally, in Step 5, histopathological and histochemical evaluations were performed on tissue samples from the rat jaws to assess changes in alveolar bone, periodontal ligament, and collagen.

To determine the potential impact of the procedures on the animals’ general health body weight was assessed weekly. The rats received anesthesia with ketamine hydrochloride 90 mg/kg and xylazine hydrochloride 9 mg/kg 14 days after the lesion was induced, and the lack of corneal reflexes was confirmed. Following that, 0.9% heparinized saline solution was perfused via the left ventricle, followed by 4% formaldehyde. The hemimandibles of the rats were then extracted from both sides using a scalpel and surgical scissors for micro-computed tomography (micro-CT) and histological investigation. Fig 1 depicts the sample description and experimental methods.

### Micro-computed tomography (micro-CT) analysis

After fixing the hemimandibles in 4 percent formaldehyde using a liquid volume at least 30 times greater than the specimen, we subjected them to micro-CT scanning (Shimadzu Corp., Kyoto, Japan; MicroCT.SMX-90 CT) with 360o rotation, 70kV intensity, and 100 mA. The images were then reconstructed using inspeXio SMX-90CT software (Shimadzu Corp., Kyoto, Japan) with a voxel size of 10μm and a resolution of 1024x1024, resulting in 541 images per sample. The Digital Imaging and Communications in Medicine (DICOM) file format was used to export all datasets.

We have used the RadiAnt DICOM Viewer 5.0.1 (Medixant, Poznan, Poland) to evaluate the three-dimensional (3D) reconstruction of the hemimandibles. The 3D models were positioned in standardized orientations, facilitating the observation of the buccal and lingual tooth faces. In this study, the vertical levels of the alveolar bone were assessed through the measurement of the distance between the cementum-enamel junction (CEJ) and the alveolar bone crest (ABC) at six specific points on the first inferior molar. These points included the mesiobuccal, buccal, distobuccal, distolingual, lingual, and mesiolingual regions. The measurements obtained from these points were subsequently averaged in millimeters (mm) [23].

We have utilized CTAn software (v.1.15) to evaluate alveolar bone quality using a dataset of 60 images taken from the region of the lower first molar bone. The region of interest (ROI) was then expanded to include the furcal region between the roots of the teeth. An examiner manually outlined the bone in each coronal plane, starting from the nearest point to the vestibular root and extending to the farthest point from the lingual root. To ensure accurate and consistent measurements in micro-CT, we first performed a calibration to verify the consistency of ROI size. Specifically, the evaluator scanned the same animal at least three times. This repetitive scanning approach provided multiple datasets for comparison, thus allowing for the identification of any variations in ROI size. By analyzing the ROI across multiple scans of the same animal, the evaluator confirmed that the ROI size remained consistent.

The CTAn software followed the manufacturer’s recommended threshold adjustment procedure to distinguish between cortical bone, trabecular bone, and bone marrow. It segmented the various gray color scores in the image using a threshold range of 31–71. Subsequently, the following parameters were measured on the remaining unaffected bone: residual bone volume (BV), percentage of bone volume (BV/TV), trabecular thickness (Tb.Th), trabecular number (Tb.N), and trabecular spacing (Tb.Sp). Assessors of micro-CT data were unaware of group distribution.

### Histopathological and histochemical evaluation

The researchers collected the hemimandibles of each specimen and subjected them to a 24-hour post-fixation procedure using a 4% formaldehyde solution. After that, they underwent a 90-day demineralization process using a 10% ethylenediaminetetraacetic acid (EDTA) solution. Afterward, we dehydrated the fragments with alcohol, followed by diaphanization in xylene, and finally embedded them in paraplast. Once we completed the embedding process, we sliced the specimens using a Leica RM 2045 microtome (Leica Microsystems, Nussloch ‐ Germany) to obtain 5 μm-thick slices oriented mesiodistally. Then, we individually mounted these resulting sections on slides. The staining procedure we employed in this study involved applying hematoxylin and eosin (H&E) to the designated sections. Subsequently, we took photomicrographs using a digital color camera (Nikon Eclipse E200, Tokyo, Japan) attached to an optical microscope (Leica QWin Plus ‐ Leica Microsystems, Nussloch ‐ Germany).

We assessed the inflammatory features of the periodontal tissues by examining semi-serial sections extending the entire length of the mandible. We evaluated the severity of the disease based on the extent, intensity, and character of the inflammatory infiltration, as well as the integrity of the bone. We evaluated the intensity of inflammatory infiltrate, categorizing it as absent, minimal, moderate, or extensive. The extent of inflammatory infiltrate was assessed, recording its presence as absent, involving a portion of the furcation area connective tissue, or involving the entire furcation area connective tissue.

Additionally, we stained sections of alveolar bone with PicroSirius Red using the modified protocol from Chemelo et al. [27] and observed them under a polarized light microscope at 40x magnification to evaluate the collagen content of the alveolar bone. We employed ImageJ software to calculate the collagen total area and perimeter of collagen fibers for each sample. We determined the collagen content by averaging the threshold percentages from three fields/sections. We measured the total area and dimensions of collagen fibers in the alveolar bone and expressed them in μm^2^. The analysts performed all the analyses without knowledge of the group allocation.

### Statistical analyses

We conducted the statistical analyses using GraphPad Prism 9.0 software (GraphPad, San Diego, CA, USA). The normality of data distribution was assessed using the Shapiro-Wilk test. Subsequently, one-way ANOVA was employed for statistical analysis, followed by the Tukey test for post hoc comparisons. Bodyweight data were analyzed using repeated-measurement two-way ANOVA. All data are presented as mean ± standard error of the mean (SEM), and statistical significance was considered at p < 0.05. For histopathological examination, a descriptive analysis was performed.

## Results

### Bodyweight evaluation

**Fig 2:**
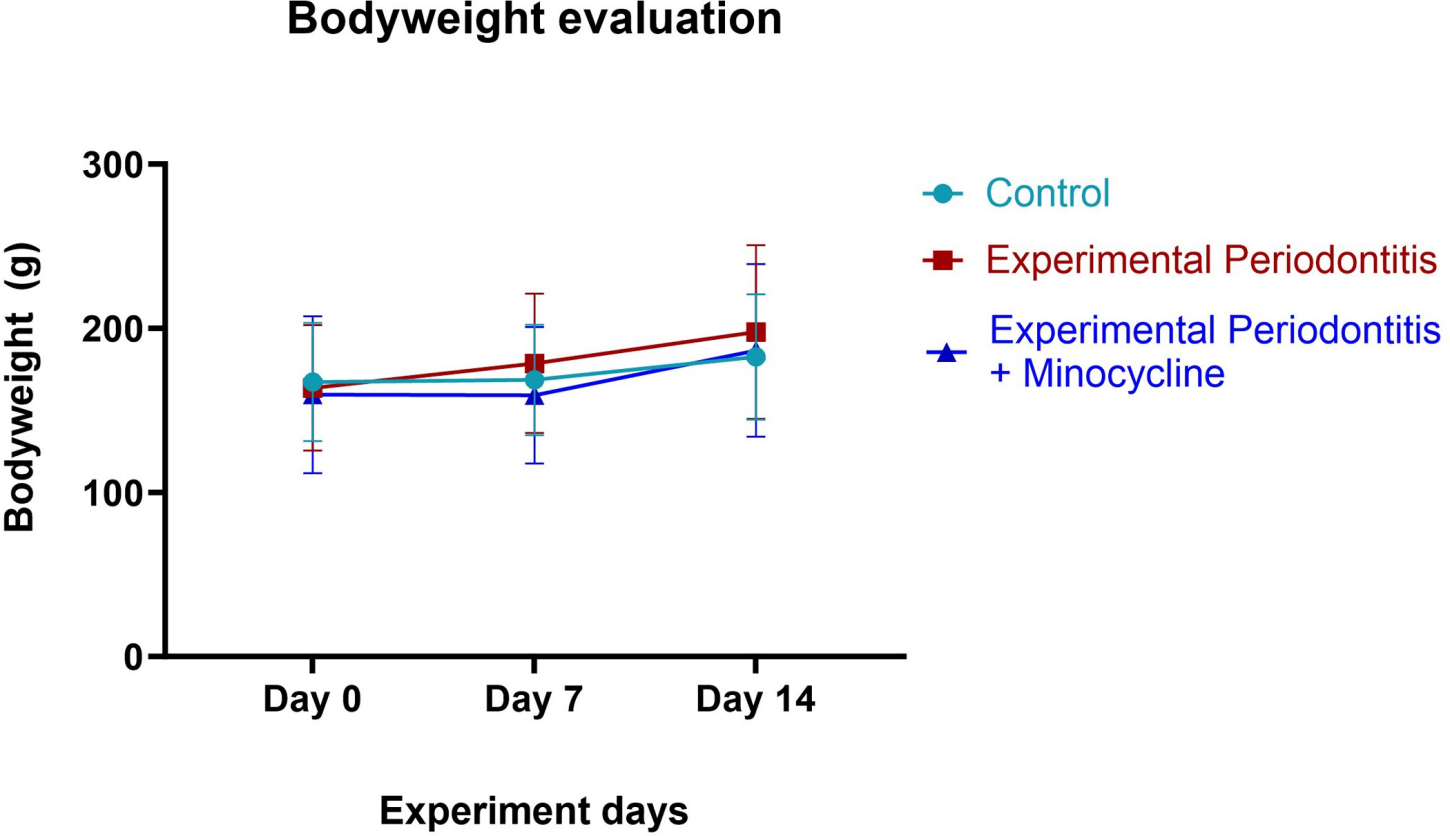
Mean body weight of different groups at baseline and along four weeks of experimental procedures. The whiskers represent the standard error of the mean.

The present investigation found no statistically significant differences in mean body weight among groups, which remained steady during the entire evaluation period (p>0.05). Animals within each group demonstrated comparable bodyweight trends, suggesting that the administered therapies did not exert a substantial influence on their general health, growth, or weight acquisition (Fig 2).

### Micro-computed tomography (micro-CT) analysis

#### Minocycline treatment reduces alveolar bone resorption in Wistar rats with Experimentally Induced periodontitis

**Fig 3:**
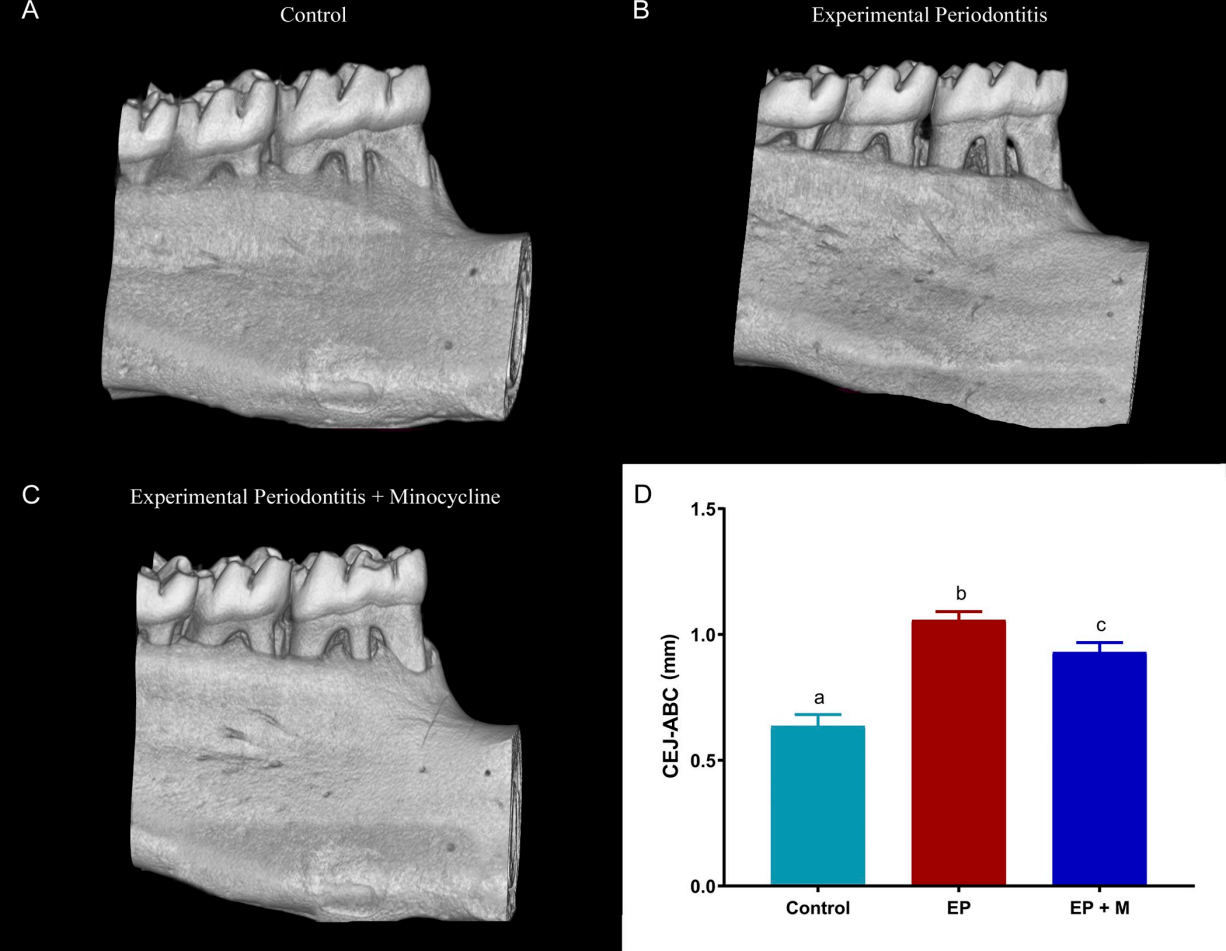
The 3D reconstructions of the hemimandibles for control, experimental periodontitis (EP), and experimental periodontitis + minocycline (EP+M) groups are shown in Images A, B, and C. The red lines indicate the distance between the cementum-enamel junction (CEJ) and the alveolar bone crest (ABC) at distolingual, lingual, and mesiolingual regions. The graph shows the statistical results and distinct letters indicate significant differences (p < 0.05). The statistical analysis involved ANOVA, followed by the Tukey post hoc test (n = 10 per group).

Analysis of hemimandibles revealed that animals in the periodontitis group (1.1mm ± 0.03) exhibited significantly greater bone loss compared to the control group (0.64mm ± 0.05; p<0.0001). In contrast, animals treated with minocycline (0.93mm ± 0.04) demonstrated a remarkable reduction in alveolar bone resorption compared to the EP group (p<0.05) although it remained slightly higher than the control group (p = 0.0002), depicted in Fig 3.

#### Minocycline treatment modulates bone microstructure in Wistar rats with experimentally induced periodontitis

**Fig 4:**
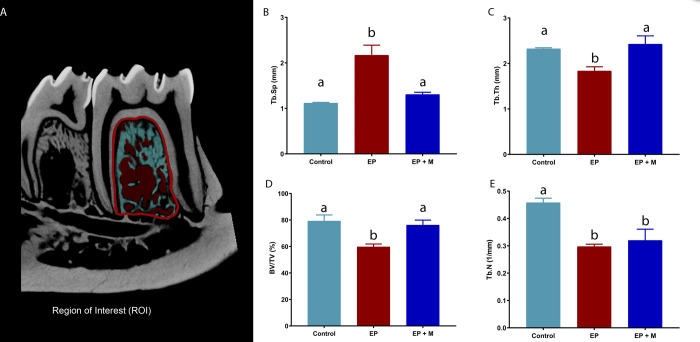
Micro-CT quality analysis was performed using CTAn software. The micro-CT quality analysis was executed with CTAn software, focusing on the region of interest, namely, the furcation region—the bone situated between the roots of the tooth. Parameters measured included trabecular spacing (Tb.Sp), trabecular thickness (Tb.Th), bone volume to total tissue volume ratio (BV/TV), and trabecular number (Tb.N). Groups with the same letters indicate no significant differences (p > 0.05). Statistical analysis involved ANOVA followed by the Tukey post hoc test (n = 10 per group).

Based on the diagnosed bone loss, the bone quality of the remaining alveolar bone was assessed. Trabecular spacing (Tb.Sp) exhibited a significant increase in the periodontitis group (2.2mm ± 0.22) compared to the control group (1.1mm ± 0.01; p = 0.0002). Conversely, the administration of minocycline significantly reduced Tb.Sp (1.3mm ± 0.05) when compared to untreated animals (p = 0.0015), bringing it to levels similar to those of the control group (Fig 4).

The EP group demonstrated a noteworthy reduction (1.8mm ± 0.09) in the trabecular thickness parameter (Tb.Th) in comparison to the control animals (2.3mm ± 0.02; p = 0.02). In contrast, periodontitis with minocycline treatment led to an increase in thickness (2.4mm ± 0.18) compared to the untreated group (p = 0.009).

Regarding the bone volume percentage (BV/TV), the EP group exhibited a substantial decrease (60% ± 2.1) when compared to the control animals (79% ± 4.5; p = 0.006). It is worth noting that the administration of minocycline resulted in a significant increase in BV/TV (76% ± 3.7) compared to the untreated group (p = 0.01). However, no significant difference was observed between the minocycline-treated group and the control group.

Finally, EP led to a reduction (0.3 1/mm ± 0.008) in the number of trabeculae (Tb.N) in comparison to the control group (0.46 1/mm ± 0.02; p = 0.002). Conversely, it was noted that the administration of minocycline did not result in a significant difference (0.32 1/mm ± 0.04) when compared to the group affected by periodontitis (p = 0.81). The complete data description is available in the S1 and S2 Tables.

### Histopathological and histochemical evaluation

#### Minocycline treatment reduced bone loss and preserved the periodontal ligament

**Fig 5:**
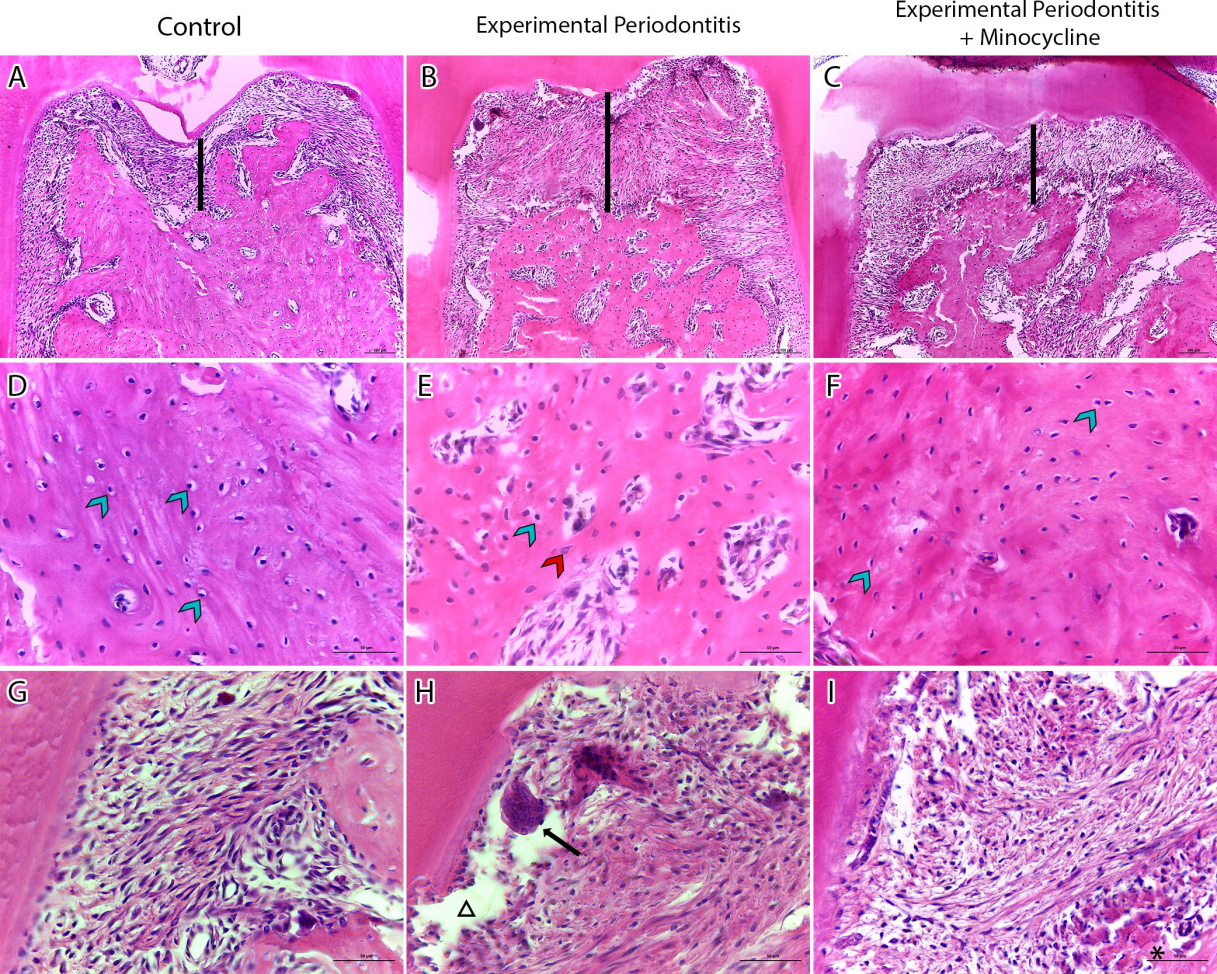
Illustrative 100 μm-scale photomicrographs of 10x magnification A, B, and C display the furcation area of the control, experimental periodontitis, and experimental periodontitis with minocycline groups, respectively. The black bold lines represent the distance from the pulp chamber floor to the alveolar bone. Photomicrographs D, E, and F provide a higher magnification with a 50 μm-scale view of the furcation area. Blue arrowheads indicate osteocytes, while red arrowheads indicate lacunae without osteocytes. Photomicrographs G, H, and I display the periodontal ligaments at 40x magnification with a 50 μm scale, representing the control, experimental periodontitis, and experimental periodontitis with minocycline groups, respectively. In image H, the triangle indicates the area of discontinuity of periodontal ligament fibers and the black arrow points to foreign body giant cells. In image I, the black asterisk indicates the preservation of alveolar bone compared to the experimental periodontitis group.

For histological assessment, we focused on the furcation region, specifically the alveolar bone and periodontal ligament between the roots (Fig 5). In the control group, the periodontal ligament remained intact, and no inflammation was observed (Fig 5A, 5D and 5G). In contrast, the EP group exhibited inflammatory infiltration, a pronounced loss of the alveolar bone (Fig 5B), lacunae without osteocytes (Fig 5E), areas of periodontal ligament discontinuity, disorganization of periodontal ligament fibers, and the presence of foreign body giant cells (Fig 5H). These histological findings corroborate the micro-CT results, which showed a decrease in trabecular thickness and number in the EP group. In the EP+M group, we observed significant preservation of the periodontal ligament, and consequently, the alveolar bone in the furcation region (Fig 5C and 5F). There was a modest presence of inflammatory infiltration within the alveolar bone (Fig 5C) and an increased organization of periodontal ligament fibers (Fig 5I). These results align with the micro-CT findings, which indicated increased BV/TV and reduced trabecular spacing.

### Minocycline treatment led to an increase in the amount and dimension of collagen fibers within the alveolar bone

**Fig 6:**
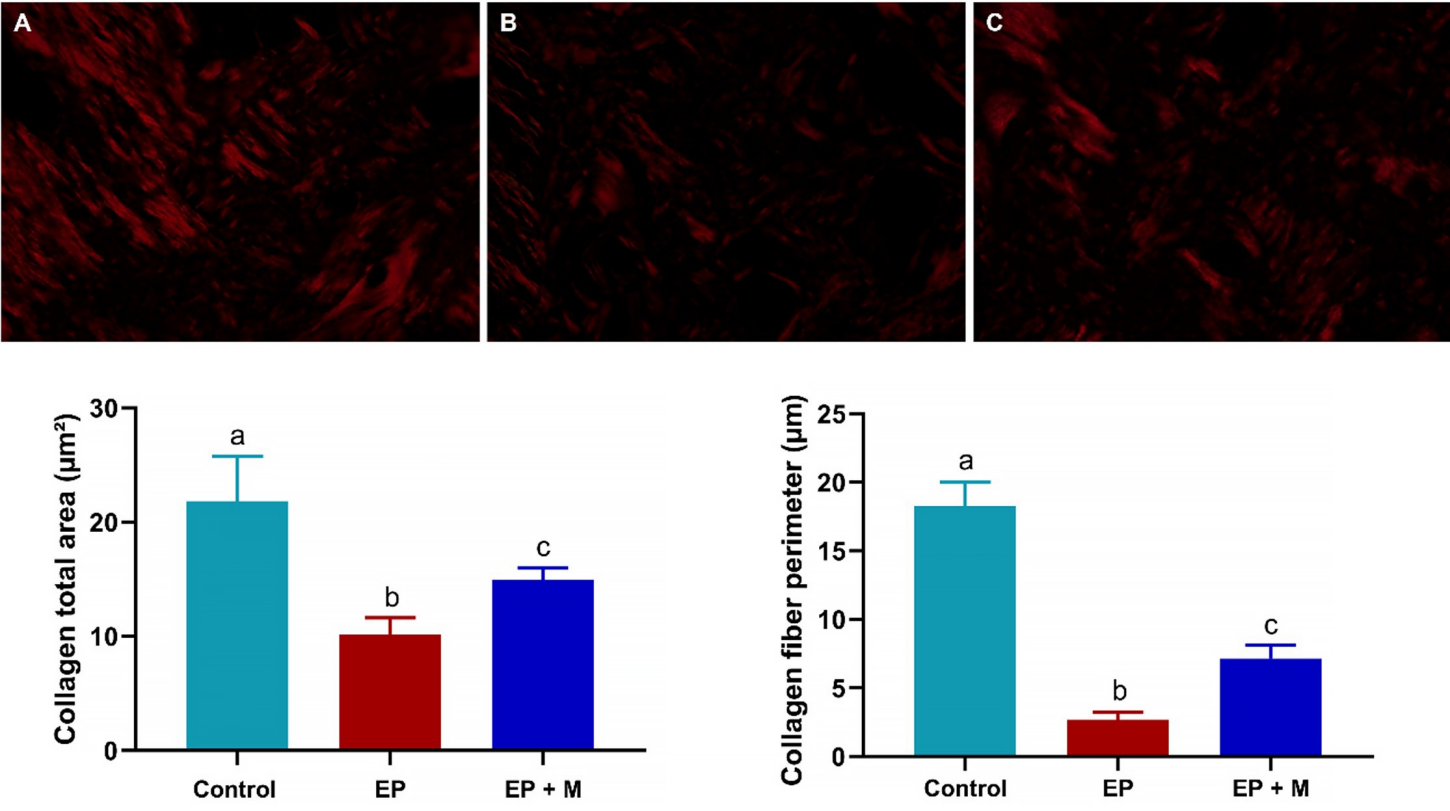
Alveolar bone collagen content and fiber dimensions were assessed through Picrosirius red. Figure A represents the control group, Figure B represents the EP group, and Figure C the EP+M group. The periodontitis group had reduced collagen content and thinner fibers compared to the control group. Minocycline-treated animals showed increased collagen content and thicker fibers, with statistical significance determined by one-way ANOVA with Tukey’s post-test.

Regarding the bone collagen content, the periodontitis group exhibited a reduction compared to the control group (333760 μm^2^ ± 66136 vs. 855192 μm^2^ ± 151204; p<0.0001). In contrast, animals treated with minocycline showed a significant increase in remaining alveolar bone collagen compared to the EP group (684552 μm^2^ ± 156576 vs. 333760 μm^2^ ± 66136; p = 0.0635), with no significant difference from the control group (855192 μm^2^ ± 151204; p = 0.0003; Fig 6). In the analysis of collagen fiber dimension, the periodontitis group demonstrated thinner collagen fibers compared to the control group (24.82 μm ± 3.617 vs. 44.63 μm ± 4.080; p<0.0001). On the other hand, the minocycline-treated animals showed a significant increase in alveolar bone fiber collagen dimension compared to the EP group (38.10 μm ± 1.028 vs. 24.82 μm ± 3.617; p<0.0001). However, this increase remained lower than that observed in the control group (44.63 μm ± 4.080; p = 0.0015), as shown in Fig 6.

## Discussion

The present study evaluated the effect of minocycline on the alveolar bone of rats with ligature-induced periodontitis. Minocycline treatment demonstrated a notable reduction in periodontal breakdown, indicating its potential effect in mitigating the damages caused by periodontitis. The assessment of bone quality further elucidated the benefits of minocycline, as evidenced by a significant reduction in trabecular spacing and an increase in trabecular thickness compared to untreated animals. Moreover, minocycline administration led to a substantial increase in bone volume percentage, approaching levels observed in the control group. The histological evaluation revealed reduced inflammatory infiltration and preserved alveolar bone crest in the treated group compared to the pronounced damage observed in the periodontitis group. Notably, minocycline treatment demonstrated its potential in preserving bone structure. These findings underscore the importance of exploring minocycline as a modulator in periodontal tissues. Furthermore, the effect of minocycline on reducing bone loss was demonstrated, highlighting its multifaceted benefits in maintaining bone integrity and collagen structure.

Regarding ligature-induced periodontitis studies design, the histological damages and bone loss kinetics have different periods varying from 3 to 28 days. The histological alterations are identified on the third to seventh day after ligature placement. Alveolar bone loss is detected on the seventh day up to the fifteenth day. After 15 days, alveolar bone loss may occur but with progression reduction that reaches stabilization in 28 or 30 days. Then, we decided to perform the within 14 days of periodontitis induction following the study of Vargas-Sanches et al. 2017 [28] with their results corroborated by Wu et al. 2020 [29]. Our research group employs this method in other studies, where we observe that leaving the ligature on the first molar for 14 days results in well-established vertical bone loss and inflammation [22–25].

It is crucial to highlight that this study is characterized by the absence of local intervention, such as root scaling and planning, that would target periodontal therapy. The primary focus is on observing the direct effects of minocycline therapy on periodontitis, especially when compared to untreated animals. The non-invasive approach, without local intervention, allows for a clearer and more specific assessment of the impacts of minocycline on the evolution of periodontitis. Understanding these isolated effects is essential before delving into comparisons with adjuvant therapies, thus avoiding potential confusion arising from multiple interventions. In addition, it should be highlighted that studies in animals are more prompt to evaluate the pathogenesis than therapeutic approaches for periodontitis. The ligature-induced periodontitis in rats is proven to mimic what happens in the mouth during the establishment of periodontitis, especially because of the retention of bacteria provided by the ligature.

Once an individual is diagnosed with a periodontal disease, a stepwise therapy strategy should be implemented depending on the current stage of the disease. This strategy should be gradual and include various interventions [5]. Adjuvant therapies are often used during the treatment phases of periodontal disease. Many antioxidants, anti-inflammatory, and antibiotic substances have been and are under study to evaluate their potential effects on reducing the severity of periodontal disease. Experimental studies often investigate these substances systemically in the absence of other treatments to determine if the drug alone can reduce inflammatory markers and, ultimately bone loss. For example, natural products like açaí [22], copaiba [24], resveratrol, curcumin [30], and *Desmarestia anceps* Montagne [31] were evaluated without any other intervention to the disease to determine their anti-inflammatory and bone-protective potential. Additionally, non-antibacterial tetracycline formulations, specifically sub antimicrobial-dose doxycycline (SDD), have also been studied and demonstrated interesting potential. They were effective in preventing and treating periodontitis and related systemic disorders. They work by blocking mammalian-derived enzymes, such as collagenases, without using antibiotics. SDD offers the advantage of a less surgical approach to periodontal care, benefiting a wider range of patients [32].

The mechanism of action behind the antibiotic properties of tetracyclines is mainly related to their ability to bind to the bacterial 30S ribosomal subunit [18]. Ribosomes facilitate protein synthesis by transforming mRNA codes into functional proteins. Prokaryotic cells have 30S and 50S subunits, while eukaryotic cells have 40S and 60S subunits. Ribosomal subunits converge at the mRNA template, allowing tRNA to transport amino acids and form proteins [33]. Therefore, minocycline prevents charged tRNA from delivering amino acids to elongate the protein chain and develop a cellular protein. This disturbance leads to a bacteriostatic effect on the prokaryotic cell, resulting in the organism’s inability to grow or reproduce [34]. Moreover, previous studies have shown that the anti-inflammatory effect of minocycline is a result of its ability to inhibit the activation and proliferation of immune cells [18]. It also reduces the levels of metalloproteinases and caspases while modulating the expression of certain genes such as Bcl-2, a mitochondrial outer membrane protein closely related to apoptosis [16,18,35].

In addition to its anti-inflammatory effects, the observed elevation in osteoblastic activity suggests a potential shift towards greater mineral deposition than resorption by osteoclasts. A study revealed that long-term exposure to minocycline at levels typically found in the plasma and gingival crevicular fluid leads to a notable increase in the quantity of active osteoblastic cells [36]. Moreover, minocycline can reduce blood monocyte migration and decrease the differentiation of monocytes into active macrophage populations [2,3]. Consequently, by limiting blood monocyte migration, the differentiation of osteoclasts is reduced. Furthermore, an in vitro study demonstrated that minocycline inhibited receptor activator of NF-κB ligand (RANKL)-induced osteoclastogenesis in bone marrow macrophages (BMMs) without affecting cell growth and phagocytic activity [4].

This could explain the findings of the present study, which not only demonstrated a reduction in vertical bone loss in the treated group but also a decrease in the intertrabecular space. Furthermore, the minocycline-treated group exhibited increased trabecular thickness, leading to a higher ratio of bone volume to tissue volume. The consistent trabecular number between groups suggests that the effect is more likely associated with the mineralization of existing trabeculae, resulting in greater thickness and overall increased bone density, as indicated by the BV/TV ratio. Although this parameter doesn’t directly measure tissue density, a study assessing rat calvaria bone reported that minocycline enhanced capillary proliferation and, consequently, osteoprogenitor cell migration into the wound area, facilitating earlier and increased mineralization and bone density in the calvarium [15].

In addition, the focused histological assessment in the furcation region provides a nuanced understanding of how minocycline influences inflammatory processes and bone preservation, offering valuable insights into the experimental periodontitis’ effects and potential therapeutic interventions. Notably, in the EP+M group, the preservation of the alveolar bone crest suggests a protective effect. The modest inflammatory infiltration indicates a controlled immune response, emphasizing the potential of the treatment in mitigating the inflammatory impact observed in the EP group.

Collagen fibers are a crucial component of alveolar bone, providing resilience and support [37]. PicroSirius red staining showed that minocycline treatment significantly increased the quantity and thickness of collagen fibers in the periapical region. This is due to minocycline’s anti-inflammatory and antimicrobial properties, which reduce inflammation and host response [36]. This environment encourages collagen synthesis, allowing fibroblasts and osteoblasts to proliferate and produce new fibers. This leads to efficient bone regeneration and mitigation of alveolar bone tissue degradation [38].

The present study has strengths and limitations. Among strengths, it should be emphasized that animal studies provide controlled situations to isolate variables. In addition, a sufficient number of animals was utilized (under the principle of reducing as much as possible, with reliable results). Also, contemporary research principles such as random allocation and blindness of the assessors were used. We also utilized several methods to refine the utilization of animal models.

Among limitations, we need to emphasize that the translation of the present findings to clinical settings is not possible. Additionally, we assessed the outcomes at only one treatment duration. Further studies are needed to investigate the effects of different treatment protocols. This study focused primarily on diagnostic aspects and did not aim to elucidate the mechanisms of action and toxicity. This is important for future research as minocycline affects various cells derived from monocytes in different organs, such as reducing the microglial population in the neural parenchyma [16] and suppressing Kupffer cells in the liver [39]. Indiscriminate use may excessively decrease these cells, potentially harming many organs. Minocycline has been associated with hepatotoxicity, including microvesicular steatohepatitis and autoimmune hepatitis-like syndromes [40]. Adverse effects of minocycline typically manifest after prolonged use, often months or years into treatment. In most cases, symptoms resolve upon drug discontinuation [40]. Therefore, further research is needed to evaluate inflammatory parameters, especially related to bone remodeling and periodontitis.

It should also be highlighted that the potential effects demonstrated herein, might generate hypotheses to prevent and treat periodontitis and also peri-implant diseases. Considering that the use of adjunctive therapies such as antimicrobial agents is preemptive in patients with systemic diseases, minocycline may provide beneficial effects regarding immune and inflammatory response control similar to other systemic antimicrobials [41,42]. Although the results are promising, the use of minocycline alone would not be justified in the dental clinic. However, our results indicate that it is essential to continue studies in this area, to understand if the potential demonstrated in the present study is proven in clinical settings.

## Conclusion

This study provides insights into the impact of systemically administered minocycline on alveolar bone under conditions of induced periodontitis. Our findings demonstrate that a 7-day minocycline treatment effectively mitigated bone damage resulting from the induced lesion, even in the absence of any other therapeutic interventions. In addition, the results clearly demonstrate benefits in parameters related to bone tissue morphology. It’s worth emphasizing that additional research is warranted to gain a more comprehensive understanding of the mechanisms underlying the interaction between this drug and bone remodeling processes.

## Supporting information

S1 Table Descriptive analysis of CEJ-ABC distance of all groups. (DOCX)

S2 Table Descriptive analysis of bone quality parameters of all groups. (DOCX)
