# Supplementary material for: Minocycline reduces alveolar bone loss and bone damage in Wistar rats with experimental periodontitis
Source: PLoS One. 2024 Oct 4;19(10):e0309390. doi: 10.1371/journal.pone.0309390 (PMC11451981; doi:10.1371/journal.pone.0309390)
Supplement: S1 Table — (DOCX) [file pone.0309390.s001.docx]

**Supplementary Materials**

**Minocycline Reduces Alveolar Bone Loss and Bone Damage in Wistar Rats with Experimental Periodontitis**

**Supplementary Table 1.** Descriptive Analysis of CEJ-ABC Distance of all Groups

| CEJ-ABC | Control | Periodontitis | Periodontitis + minocycline |
| --- | --- | --- | --- |
| Number of values | 9 | 9 | 9 |
|  |  |  |  |
| Minimum | 0.47 | 0.95 | 0.71 |
| 25% Percentile | 0.54 | 0.96 | 0.87 |
| Median | 0.68 | 1.1 | 0.96 |
| 75% Percentile | 0.71 | 1.1 | 0.97 |
| Maximum | 0.73 | 1.3 | 1.1 |
|  |  |  |  |
| Mean | 0.64 | 1.1 | 0.93 |
| Std. Deviation | 0.1 | 0.1 | 0.11 |
| Std. Error of Mean | 0.046 | 0.033 | 0.038 |
|  |  |  |  |
| Lower 95% CI | 0.51 | 0.98 | 0.84 |
| Upper 95% CI | 0.76 | 1.1 | 1 |
