# Supplementary material for: Minocycline reduces alveolar bone loss and bone damage in Wistar rats with experimental periodontitis
Source: PLoS One. 2024 Oct 4;19(10):e0309390. doi: 10.1371/journal.pone.0309390 (PMC11451981; doi:10.1371/journal.pone.0309390)
Supplement: S2 Table — (DOCX) [file pone.0309390.s002.docx]

**Supplementary Materials**

**Minocycline Reduces Alveolar Bone Loss and Bone Damage in Wistar Rats with Experimental Periodontitis**

**Supplementary Table 2.** Descriptive Analysis of Bone Quality Parameters of all Groups

| **BV/TV** | **Control** | **Periodontitis** | **Periodontitis + minocycline** |
| --- | --- | --- | --- |
| **Number of values** | 8 | 9 | 9 |
|  |  |  |  |
| **Minimum** | 70 | 53 | 67 |
| **25% Percentile** | 71 | 55 | 70 |
| **Median** | 79 | 60 | 75 |
| **75% Percentile** | 88 | 64 | 83 |
| **Maximum** | 90 | 65 | 90 |
|  |  |  |  |
| **Mean** | 79 | 60 | 76 |
| **Std. Deviation** | 9.1 | 4.6 | 8.3 |
| **Std. Error of Mean** | 4.5 | 2.1 | 3.7 |
|  |  |  |  |
| **Lower 95% CI** | 65 | 54 | 66 |
| **Upper 95% CI** | 94 | 66 | 87 |
|  |  |  |  |
| **Tb.Th** | **Control** | **Periodontitis** | **Periodontitis + minocycline** |
| **Number of values** | 8 | 9 | 9 |
|  |  |  |  |
| **Minimum** | 2.3 | 1.7 | 2.1 |
| **25% Percentile** | 2.3 | 1.7 | 2.2 |
| **Median** | 2.3 | 1.8 | 2.3 |
| **75% Percentile** | 2.4 | 2 | 2.8 |
| **Maximum** | 2.4 | 2.1 | 3 |
|  |  |  |  |
| **Mean** | 2.3 | 1.8 | 2.4 |
| **Std. Deviation** | 0.047 | 0.18 | 0.36 |
| **Std. Error of Mean** | 0.021 | 0.089 | 0.18 |
|  |  |  |  |
| **Lower 95% CI** | 2.3 | 1.6 | 1.9 |
| **Upper 95% CI** | 2.4 | 2.1 | 3 |
|  |  |  |  |
| **Tb.N** | **Control** | **Periodontitis** | **Periodontitis + minocycline** |
| **Number of values** | 9 | 9 | 9 |
|  |  |  |  |
| **Minimum** | 0.42 | 028 | 0.22 |
| **25% Percentile** | 0.42 | 0.28 | 0.25 |
| **Median** | 0.47 | 0.3 | 0.32 |
| **75% Percentile** | 0.49 | 0.32 | 0.4 |
| **Maximum** | 0.49 | 0.32 | 0.42 |
|  |  |  |  |
| **Mean** | 0.46 | 0.3 | 0.32 |
| **Std. Deviation** | 0.036 | 0.017 | 0.082 |
| **Std. Error of Mean** | 0.016 | 0.0085 | 0.041 |
|  |  |  |  |
| **Lower 95% CI** | 0.41 | 0.27 | 0.19 |
| **Upper 95% CI** | 0.5 | 0.32 | 0.45 |
|  |  |  |  |
| **Tb.Sp** | **Control** | **Periodontitis** | **Periodontitis + minocycline** |
| **Number of values** | 8 | 9 | 9 |
|  |  |  |  |
| **Minimum** | 1.1 | 1.7 | 1.2 |
| **25% Percentile** | 1.1 | 1.8 | 1.2 |
| **Median** | 1.1 | 2.1 | 1.3 |
| **75% Percentile** | 1.2 | 2.6 | 1.4 |
| **Maximum** | 1.2 | 2.7 | 1.4 |
|  |  |  |  |
| **Mean** | 1.1 | 2.2 | 1.3 |
| **Std. Deviation** | 0.032 | 0.44 | 0.094 |
| **Std. Error of Mean** | 0.014 | 0.22 | 0.047 |
|  |  |  |  |
| **Lower 95% CI** | 1.1 | 1.5 | 1.2 |
| **Upper 95% CI** | 1.2 | 2.9 | 1.5 |
|  |  |  |  |
